# Exposure to Community Violence, Affiliations With Risk-Taking Peer Groups, and Internet Gaming Disorder Among Chinese Adolescents: The Moderating Role of Parental Monitoring

**DOI:** 10.3389/fpsyg.2019.02074

**Published:** 2019-09-20

**Authors:** Qiao Liang, Chengfu Yu, Quanfeng Chen, Xiaodong Xie, Han Wu, Jintao Xing, Shihua Huang, Kai Dou

**Affiliations:** ^1^Department of Psychology, School of Education, Guangzhou University, Guangzhou, China; ^2^School of Education, Research Center of Adolescent Psychology and Behavior, Guangzhou University, Guangzhou, China; ^3^Human Resources Department, South China Normal University, Guangzhou, China; ^4^School of Psychology, South China Normal University, Guangzhou, China; ^5^Department of Psychology, School of Economics and Management, Guangzhou University of Chinese Medicine, Guangzhou, China

**Keywords:** adolescents, exposure to community violence, Internet gaming disorder, affiliations with risk-taking peer groups, parental monitoring

## Abstract

Among adolescents, exposure to community violence (ECV) has been consistently linked to problem behaviors such as Internet gaming disorder (IGD). However, the associated risk and protective factors have not been adequately explored in past studies. Therefore, in accordance with the risk-buffering model and social development model, this study aimed to test whether parental monitoring moderated the relationship between ECV and IGD among adolescents, and whether this moderating effect was mediated by affiliations with risk-taking peer groups. A sample of 2,423 Chinese middle-school students anonymously responded to questionnaires that assessed ECV, IGD, affiliations with risk-taking peer groups, and parental monitoring. The results of structural equation modeling revealed that the interaction between ECV and parental monitoring negatively related to IGD among adolescents. Specifically, the positive relationship between ECV and IGD was stronger for adolescents, who reported low levels of parental monitoring than for those who reported high levels of parental monitoring. Moreover, this moderating effect was mediated by affiliations with risk-taking peer groups. These results suggest that parental monitoring is an important protective factor that can mitigate the risk of IGD among adolescents who have been exposed to community violence. Accordingly, these findings serve as an empirical base upon which prevention and intervention strategies that are aimed at mitigating the risk of IGD among adolescents can be developed.

## Introduction

China has become the largest online gaming market in the world (CNNIC, 2019). With the rapid development of the online gaming industry, the prevalence of Internet gaming disorder (IGD) is increasing rapidly, and it has become widespread, especially among adolescents (Kuss, 2013; Griffiths et al., 2015). IGD is generally conceptualized as the physical, psychological, and social damage that is caused by the uncontrollable, excessive, and/or compulsive playing of online games (Young and de Abreu, 2011; Griffiths et al., 2015; Yu et al., 2015). IGD has serious detrimental effects on adolescents because it not only leads to a variety of externalizing problems (e.g., poor academic achievement, social disorders, problems related to sleep quality, and criminal behaviors) but also causes internalizing problems (e.g., anxiety, depression, and withdrawal) among adolescents (Henchoz et al., 2016; Zajac et al., 2017). In order to develop effective prevention and intervention strategies, it is necessary to understand the mechanisms and risk factors that are associated with IGD among adolescents.

Experience exposure to community violence is a crucial risk predictor for adolescent problem behavior (Esposito et al., 2017; Burnside et al., 2018). Exposure to community violence (ECV) refers to violence that takes place outside the home, such as neighborhoods or communities, and it can be caused by a person, who is either known or unrelated to the observer (Krug et al., 2002; Kennedy and Ceballo, 2014). It is characterized by witnessing or experiencing violent behaviors such as physical threats, possession of a weapon, robbery, shooting, and stabbing (Wilson and Rosenthal, 2003; Zhang et al., 2017). Adolescents may either witness violence in community public areas or become victims of community violence (Fowler et al., 2009). Based on the general strain theory (Agnew, 2001), ECV might serve as a stressor that increases adolescents’ negative emotions like anxiety, fear, and anger, which, in turn, may lead to them turning to online games as coping strategies to alleviate their negative emotions. There is considerable evidence suggesting that ECV significantly predicts various addictive behaviors among adolescents (Wright et al., 2013), especially IGD (Park et al., 2008; Yu et al., 2017). For instance, based on latent profile analysis examining the interaction of multiple environmental factors on adolescent IGD in a 6-month longitudinal study, Yu et al. (2017) found that ECV is an important predictor of IGD among adolescents. These findings underscore the critical role that ECV might play in increasing the risk of IGD among adolescents.

In order to maximize the effectiveness of interventions to prevent the negative effects of ECV, this study aimed to explore the moderators and mediators that underlie this relationship. Specifically, based on the risk-buffering model (Luthar et al., 2015) and social development model (Hawkins and Weis, 1985), we examined whether parental monitoring moderates the relationship between ECV and IGD, and whether this moderating effect is mediated by adolescent affiliations with risk-taking peer groups.

### The Moderating Role of Parental Monitoring

Although ECV is a crucial risk factor for IGD among adolescents, there are individual differences in the influence that ECV has on IGD. According to the risk-buffering model (Luthar et al., 2015), family protective factors serve as one of the essential aspects of resiliency of adolescents, who are facing adversity. Positive family factors (e.g., parental monitoring) could attenuate the adverse effects of living in a high-risk community on adolescents’ problem behaviors. Parental monitoring refers to a set of parental behaviors that include tracking and staying informed about the whereabouts, activities, and companions of adolescents (Dishion and McMahon, 1998). Parental monitoring refers to behaviors that involve “keeping tabs” on where, how, and who with adolescents spend their leisure activities, which has been shown to largely reduce the likelihood of adolescents engaging in or developing risky behaviors (Kliewer et al., 2006; Vaala and Bleakley, 2015). From the autonomy-granting perspective, parental monitoring could lead to feelings of being over-controlled and having weakened autonomy among adolescent (Steinberg and Silverberg, 1986; Laird et al., 2003). However, parental monitoring has been shown to vary in different social contexts (Luthar et al., 2015) so that in high-risk environment (e.g., ECV), effective parental monitoring might see as a way of demonstrating care and concern for adolescent children (Dishion and McMahon, 1998; Luthar et al., 2015). For example, in areas of armed conflict, parental monitoring can improve the mental health of adolescents, who have been exposed to violence (Tol et al., 2013).

Abundant empirical studies have established that adolescents of parents who display poor parental monitoring behaviors are more likely to exhibit problem behaviors (e.g., IGD, Internet abuse) than their counterparts whose parents engage in greater parental monitoring behaviors (Ding et al., 2017; Su et al., 2018). Bacchini et al. (2011) reported that parental monitoring was a moderator that minimized the negative effects of ECV on adolescent antisocial behaviors, anxiety, and depression. Similarly, Low and Espelage (2014) found that parental monitoring mitigated the effects of ECV on adolescent delinquency. Therefore, we proposed the following hypothesis:

*Hypothesis 1*: Parental monitoring would moderate the relationship between ECV and IGD among adolescents. Specifically, the adverse effects of ECV on IGD would be stronger among adolescents, whose parents exhibit low rather than high levels of parental monitoring.

### The Mediating Role of Affiliations With Risk-Taking Peer Groups

The role that affiliations with risk-taking peer groups play in adolescent development has received significant attention in recent decades. An affiliation with risk-taking peer groups refers to adolescent associations with peers who engage in risk-taking behaviors such as fighting, stealing, and substance use (Saxbe et al., 2015; Elam et al., 2017). According to the social development model (Hawkins and Weis, 1985), socialization units (e.g., family and community) have significant influence on adolescent’s behaviors in the process of their social development, and these influences always function through peer socialization processes. If adolescents establish negative social bond in violent community environment, they are more likely to be affiliated with deviant peers, in turn, develop deviant behaviors (i.e., IGD). In other words, ECV might increase the likelihood of adolescents’ affiliation with risk-taking peers groups and the consequent development of IGD.

On the one hand, risk-taking peer groups are more likely to be found in violent rather than nonviolent communities (Low and Espelage, 2014). A violent community may influence adolescents to change their normative codes of conduct; as a result, they may become affiliated with risk-taking peer groups. For instance, Goodearl et al. (2014) found that ECV significantly increased the likelihood of adolescents’ affiliations with risk-taking peers. On the other hand, affiliations with risk-taking peer groups may be an essential predictor of IGD among adolescents (Wang et al., 2015; Zhu et al., 2015; Tian et al., 2019). Specifically, risk-taking peer groups may strengthen adolescents’ positive attitudes and expectations about problem behaviors (e.g., “Internet gaming offers me more topics that I can discuss with my friends”) through observational learning, peer norms, and peer pressure (Iwamoto and Smiler, 2013). Considerable research has documented the mediating effect of affiliations with risk-taking peer groups on the relationship between ECV and externalizing problem behaviors among adolescents (Borofsky et al., 2013; Hou et al., 2017; Zhang et al., 2017). Therefore, we predicted that affiliations with risk-taking peer groups would mediate the relationship between ECV and IGD in adolescents.

Furthermore, the intensity and direction of the association between ECV and affiliations with risk-taking peer groups may vary depending on the level of parental monitoring. Indeed, Low and Espelage (2014) found that parental monitoring mitigated the adverse effects of ECV on peer victimization, which is an important predictor of affiliations with risk-taking peer groups. By contrast, low levels of parental monitoring may precipitate adolescents joining a deviant peer group, which may result in an increased likelihood of developing an addiction to the Internet (Ding et al., 2017). Parents can create a normative environment for adolescents through care, guidance, and reinforcement, whereby adolescents are required to conform to parental expectations; consequently, parental monitoring may decrease opportunities for affiliations with risk-taking peer groups and suppress risk behaviors such as IGD (Su et al., 2018). Wang et al. (2014) also reported that parental monitoring could attenuate the adverse effects of deviant peer affiliation on adolescent problem behaviors. Therefore, we proposed the following hypothesis:

*Hypothesis 2*: The moderating effect of parental monitoring on the relationship between ECV and IGD among adolescents would be mediated by affiliations with risk-taking peer groups.

### The Present Study

**Figure 1:**
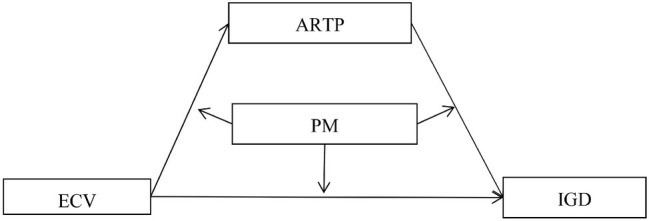
The proposed mediated moderation model. Note: ARTP, affiliations with risk-taking peer groups; ECV, exposure to community violence; IGD, Internet gaming disorder; PM, parental monitoring.

Based on the risk-buffering model and social development model, the present study aimed to explain the mechanisms by which three microcosmic systems, namely, communities, families, and peers, influence IGD among adolescents. Specifically, we sought to test (1) whether parental monitoring moderated the direct relationship between ECV and IGD and (2) whether affiliations with risk-taking peer groups mediated this moderating effect. Figure 1 illustrates the proposed research model.

## Method

### Participants

In this study, participants were selected from four junior middle schools in the Guangdong province, southern China, using stratified and random cluster sampling techniques. The sample was first stratified by region (medium versus large cities); then stratified by school type (public versus private schools). Random cluster sampling was used to randomly choose five classes in each grade of each school. A total of 2,423 adolescents (males = 50.76%, *n* = 1,230) between the ages of 9 and 16 years (*M* = 11.63, SD = 1.54) participated in the study.

### Procedure

Data were collected from participants by a well-trained psychology professor and graduate students during class hours. Written informed consent was obtained from participants, teachers, and parents before the commencement of data collection. Participants were provided with a full description of the present study; subsequently, they were informed that they must independently complete all the questionnaire items and they could choose not to answer any question that made them feel uncomfortable. The data collector also emphasized the confidentiality of the collected responses. Participants were compensated with a pencil once they completed the survey. In addition, the ethics in human research committee of the School of Education, Guangzhou University, and School of Psychology, South China Normal University approved all test materials and survey procedures that were used in this study.

### Measures

Data were collected using the ECV Questionnaire, Parental Monitoring Questionnaire, Affiliations with Risk-Taking Peer Groups Questionnaire, IGD Questionnaire, and Impulsivity Scale.

#### Exposure to Community Violence

ECV was measured using the Chinese version of the ECV Questionnaire (Zhang et al., 2017). This questionnaire consists of six items, each of which requires the respondent to indicate how often they have witnessed six types of violence in their neighborhood during the past 6 months (e.g., people were robbed) on a 5-point scale ranging from “never” (score = 1) to “always” (score = 5). The mean of the six items is calculated to yield a composite score; high scores indicate a high level of ECV. In this study, Cronbach’s alpha was 0.73 for this scale.

#### Parental Monitoring

Parental monitoring was measured using the Chinese version of the Parental Monitoring Questionnaire (Deng et al., 2018). The questionnaire consists of 21 items that assess parental knowledge, expectations, and supervision. Respondents are required to indicate the frequency with which they experience parental monitoring on a 5-point scale that ranges from “never” (score = 1) to “always” (score = 5). Individual item scores recorded on the 21 items are averaged to yield a composite score; high scores indicate high levels of parental monitoring. In this study, Cronbach’s alpha of the scale was 0.76.

#### Affiliations With Risk-Taking Peer Groups

Adolescent affiliation with risk-taking peers groups was measured with 10 items adapted from prior published questionnaires (Kendler et al., 2007; Li et al., 2013; Zhu et al., 2015). Adolescents indicated how many of their close friends had consumed alcohol, smoked cigarettes, gambled, engaged in online dating, sexual intercourse, and online gaming, gotten into fights, stolen goods, used drugs, and committed robbery or extortion through an Affiliations with Risk-Taking Peer Groups Questionnaire. Their responses are recorded on a 5-point scale ranging from “none of them” (score = 1) to “6 or more” (score = 5). Individual item scores recorded across the 10 items are averaged to yield a composite score; high scores indicate greater affiliations with risk-taking peer groups. In this study, Cronbach’s alpha of this scale was 0.86.

#### Internet Gaming Disorder

IGD was assessed using the Chinese version of the IGD Questionnaire (Yu et al., 2015). It consists of 11 items, each of which requires the respondent to report the degree to which they have experienced the symptoms of IGD in the past 6 months (e.g., “Have you spent more time playing online games than was planned?”) on a 3-point scale: 0 = never, 0.5 = sometimes, and 1 = yes. Individual item scores across all 11 items are averaged to yield a composite score; higher scores indicate greater severity of IGD. In this study, Cronbach’s alpha of this scale was 0.79.

#### Control Variables

Given that prior studies have shown that gender, age, and impulsivity are associated with IGD among adolescents (Gentile, 2009; Hyun et al., 2015; Lee and Mckenzie, 2015), we included them as control variables in the statistical models. Impulsivity was assessed using the Urgency, Premeditation, Perseverance, Sensation Seeking, and Positive Urgency (UPPS-P) Scale (Cyders et al., 2014). In this study, this scale demonstrated excellent internal consistency (Cronbach’s *α* = 0.76).

### Statistical Analyses

Version 25.0 of the Statistical Package for the Social Sciences was used to compute correlations and descriptive statistics. Further, we conducted structural equation modeling using version 7.1 of Mplus to examine the mediating and moderating effects of the respective variables (Muthén and Muthén, 1998/2012). Goodness-of-fit for the model was assessed using several standard indices, including *χ*^2^/df < 5, CFI > 0.90, TLI > 0.90, IFI > 0.90, RMSEA <0.08, P-CLOSE > 0.05, and SRMR < 0.09 (Hoyle, 2012).

## Results

### Preliminary Analyses

Adolescents who responded to any item on the ECV and Affiliations with Risk-Taking Peer Groups Questionnaires with a response greater than “never” were considered to have experienced ECV and affiliations with risk-taking peers, respectively. According to this standard, the prevalence of ECV and ARTP are 74.33 and 47.34%, respectively. Moreover, according to Young and de Abreu (2011), the total score of IGD questionnaire ≥5 was considered as an indicator of having IGD. Therefore, 2.97% (*n* = 72) of the participants in the present sample displayed IGD, which is consistent with national Chinese adolescent data (Dong and Lin, 2011) and recent literature (Yu et al., 2015).

The means, standard deviations, and correlation coefficients that were computed for all variables of the present study are displayed in Table 1. The results show that ECV and affiliations with risk-taking peer groups were positively related to IGD and that parental monitoring was negatively related to IGD. These findings suggest that ECV, low levels of parental monitoring, and high affiliations with risk-taking peer groups are potential risk factors for IGD among adolescents; similarly, ECV and low levels of parental monitoring are potential risk factors for affiliations with risk-taking peer groups.

**Table 1:** Descriptive statistics and correlations for all variables.

Variables	1	2	3	4	5	6	7
1. Gender	1.00						
2. Age	0.01	1.00					
3. Impulsivity	0.07^**^	0.02	1.00				
4. ECV	−0.01	0.13^**^	0.17^**^	1.00			
5. PM	−0.09^**^	−0.02	−0.24^**^	−0.06^**^	1.00		
6. ARTP	0.07^**^	0.07^**^	0.22^**^	0.27^**^	−0.13^**^	1.00	
7. IGD	0.27^**^	0.12^**^	0.27^**^	0.18^**^	−0.19^**^	0.24^**^	1.00
Mean	0.51	11.63	2.19	1.74	2.52	1.23	1.28
SD	0.50	1.54	0.40	0.69	0.48	0.42	1.42

*Gender and age were dummy coded such that 1 = male, 0 = female; ARTP, affiliations with risk-taking peer groups; ECV, exposure to community violence; IGD, Internet gaming disorder; PM, parental monitoring*.

** *p < 0.01*.

### The Moderating Effect of Parental Monitoring on the Direct Relationship Between Exposure to Community Violence and Internet Gaming Disorder Among Adolescents

**Figure 2:**
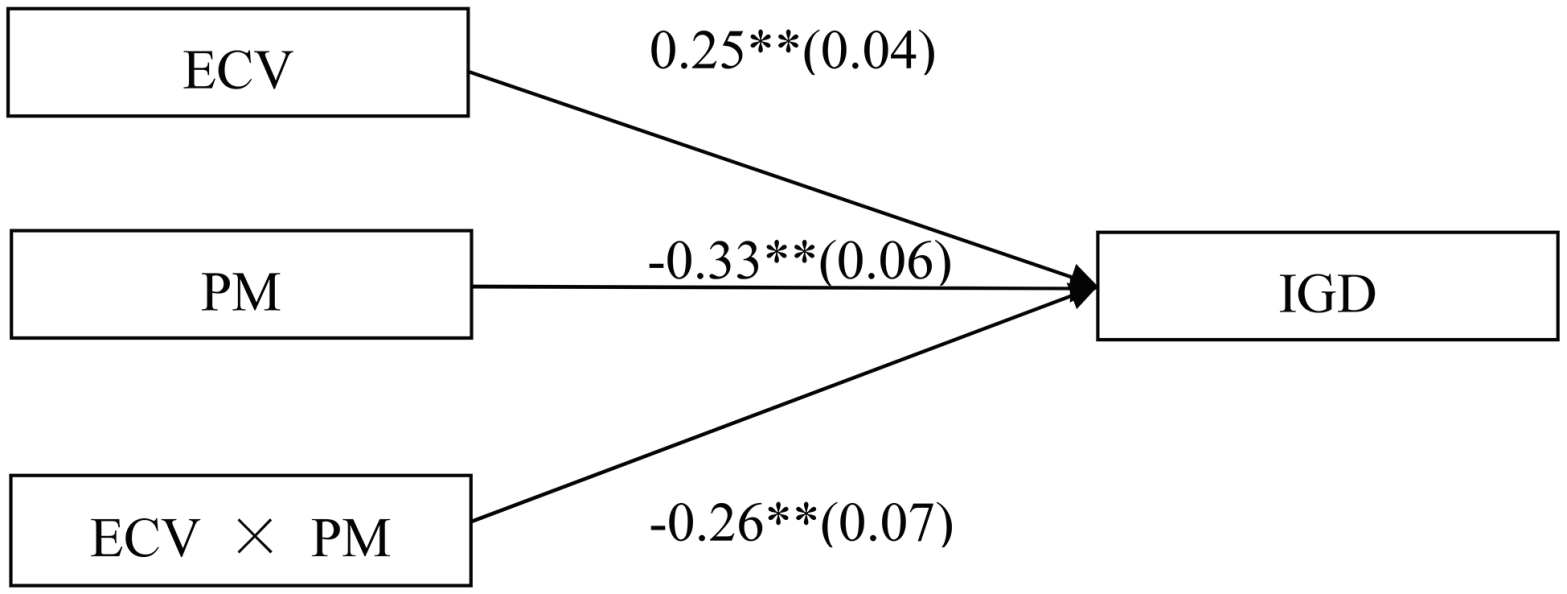
Model of the moderating role of parental monitoring on the direct relationship between exposure to community violence and IGD. Note: ECV, exposure to community violence; IGD, Internet gaming disorder; PM, parental monitoring. Values are unstandardized coefficients and standard error. Paths between gender, age, impulsivity, and each of the variables in the model are not displayed. Of those paths, the following were significant: gender (*b* = 0.69, SE = 0.05, *t* = 13.11^**^), age (*b* = 0.08, SE = 0.02, *t* = 4.86^**^), and impulsivity (*b* = 0.71, SE = 0.07, *t* = 10.34^**^) to IGD. ^*^p < 0.05, ^**^p < 0.01.

The moderated model that is presented in Figure 2 was found to be an acceptable fit for the data: χ^2^/df = 2.56, *p* < 0.05, CFI = 0.97, TLI = 0.97 IFI = 0.98, RMSEA = 0.04, P-CLOSE = 0.79, and SRMR = 0.05. The results demonstrated that the main effects of ECV (*b* = 0.25, SE = 0.04, *β* = 0.12, *t* = 6.34, *p* < 0.01) and parental monitoring (*b* = −0.33, SE = 0.06, *β* = −0.11, *t* = −5.88, *p* < 0.01) on IGD were significant. Moreover, the interaction effect between ECV and parental monitoring on IGD was also significant (*b* = −0.26, SE = 0.07, *β* = −0.06, *t* = −3.46, *p* < 0.01).

**Figure 3:**
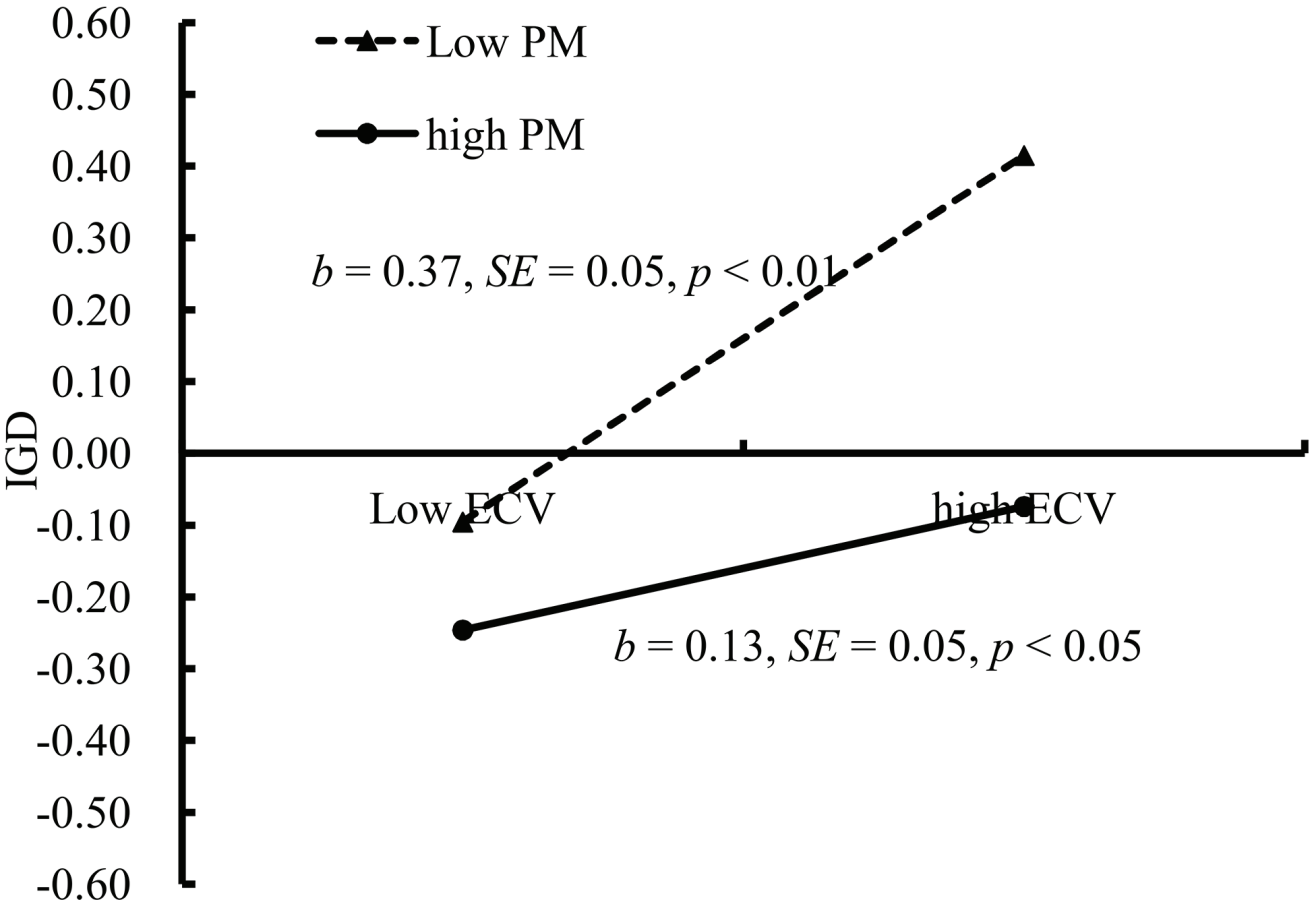
IGD among adolescents as a function of exposure to community violence and parental monitor. Note: ECV, exposure to community violence; IGD, Internet gaming disorder; PM, parental monitoring.

We conducted a simple slopes test; as depicted in Figure 3, the positive relationship between ECV and IGD was much stronger for adolescents, who reported lower levels of parental monitoring (1 SD below the *M*; *b* = 0.37, SE = 0.05, *t* = 7.35, *p* < 0.01, 95% CI [0.27, 0.47]) than for those who reported higher levels of parental monitoring (1 SD above the *M*; *b* = 0.13, SE = 0.05, *t* = 2.28, *p* < 0.05, 95% CI [0.02, 0.23]).

### The Moderating Effect of Parental Monitoring on the Indirect Relationship Between Exposure to Community Violence and Internet Gaming Disorder

**Figure 4:**
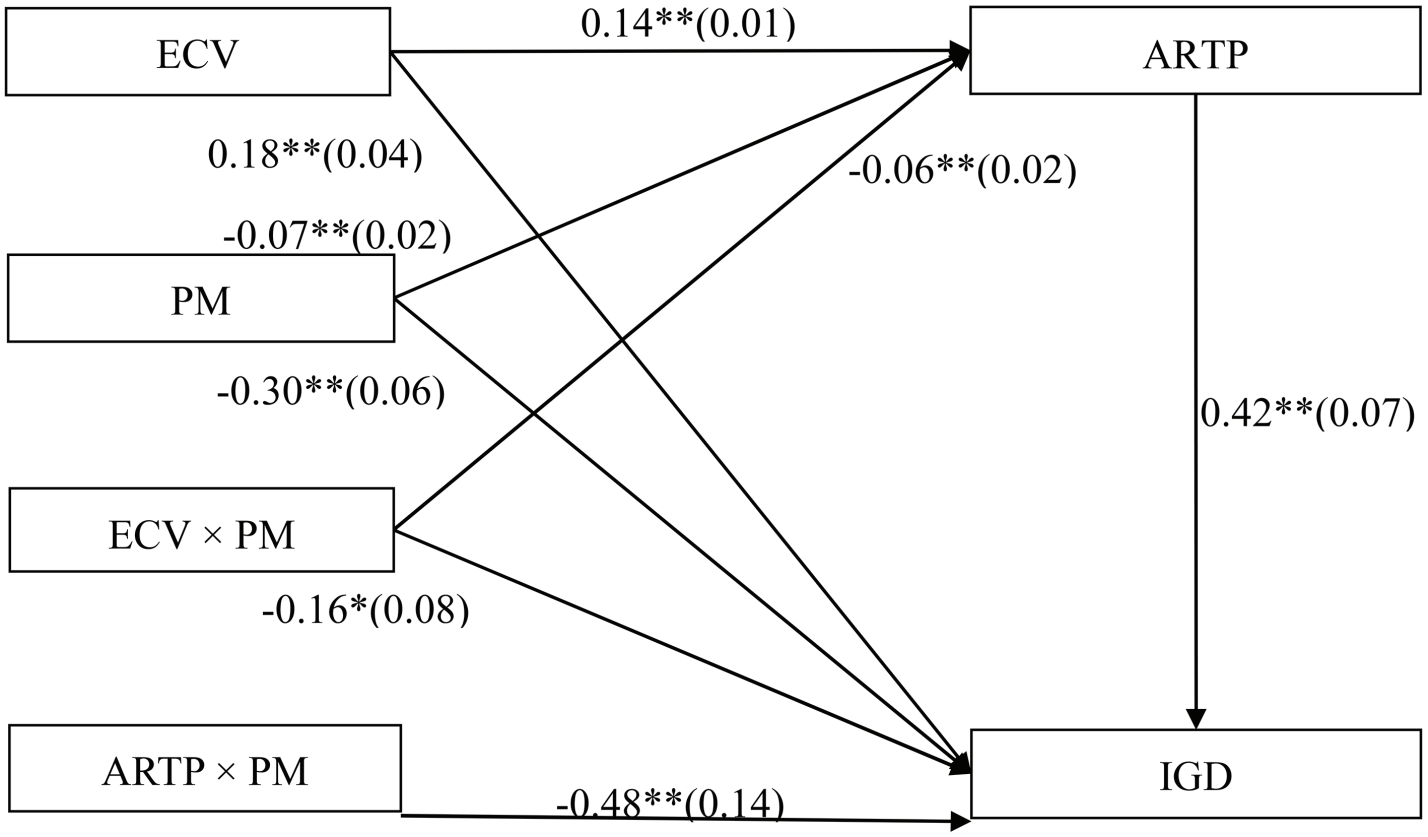
Model of the moderating role of parental monitoring on the indirect relationship between Exposure to community violence and IGD. Note: ARTP, affiliations with risk-taking peer groups; ECV, exposure to community violence; IGD, Internet gaming disorder; PM, parental monitoring. Values are unstandardized coefficients and standard error. Paths between gender, age, impulsivity, and each of the variables in the model are not displayed. Of those paths, the following were significant: gender (*b* = 0.04, SE = 0.02, *t* = 2.59^**^) and impulsivity (*b* = 0.17, SE = 0.02, *t* = 7.91^**^) to ARTP; gender (*b* = 0.67, SE = 0.05, *t* = 12.96^**^), age (*b* = 0.07, SE = 0.02, *t* = 4.40^**^), and impulsivity (*b* = 0.64, SE = 0.07, *t* = 9.28^**^) to IGD. ^*^p < 0.05, ^**^p < 0.01.

**Figure 5:**
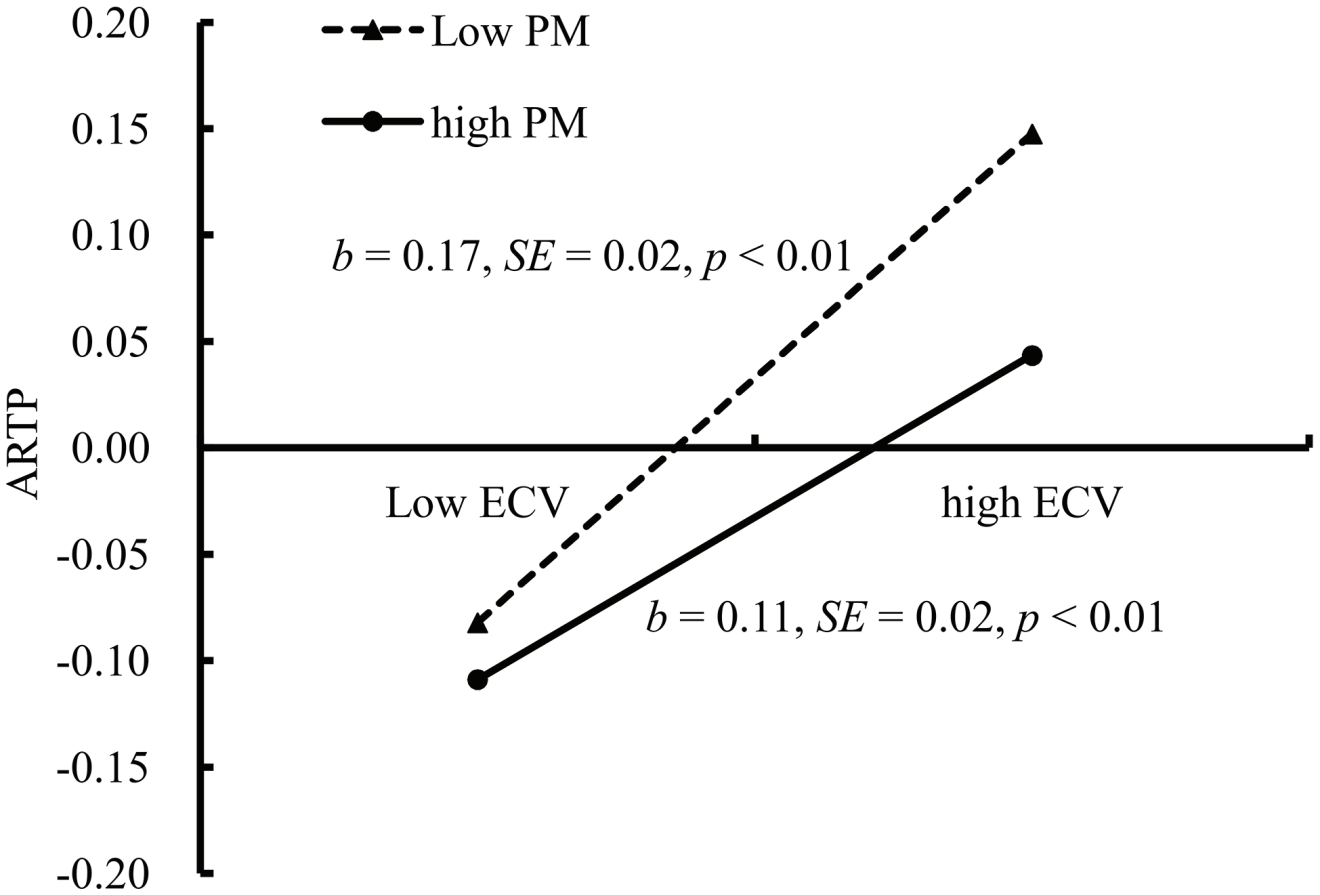
Affiliations with risk-taking peer groups among adolescents as a function of exposure to community violence and parental monitoring. Note: ARTP, affiliations with risk-taking peer groups; ECV, exposure to community violence; PM, parental monitoring.

The mediated moderation model that is presented in Figure 4 was found to be a good fit for the data: χ^2^/df = 1.87, *p* < 0.05, CFI = 0.97, TLI = 0.94, IFI = 0.96, RMSEA = 0.03, P-CLOSE = 0.65, and SRMR = 0.06. The interaction effect between ECV and parental monitoring on affiliations with risk-taking peer groups was significant (*b* = −0.06, SE = 0.02, *β* = −0.05, *t* = −2.62, *p* < 0.01). Figure 5 presents the predicted affiliations with risk-taking peer groups as a function of ECV and parental monitoring. Specifically, the positive relationship between ECV and affiliations with risk-taking peer groups was significantly stronger for adolescents, who reported lower levels of parental monitoring (1 SD below the *M*; *b* = 0.17, SE = 0.02, *t* = 10.93, *p* < 0.01, 95% CI [0.14, 0.20]) than for adolescents who reported higher levels of parental monitoring (1 SD above the *M*; *b* = 0.11, SE = 0.02, *t* = 6.65, *p* < 0.01, 95% CI [0.08, 0.14]). Thus, the relationship between ECV and affiliations with risk-taking peer groups was moderated by parental monitoring.

**Figure 6:**
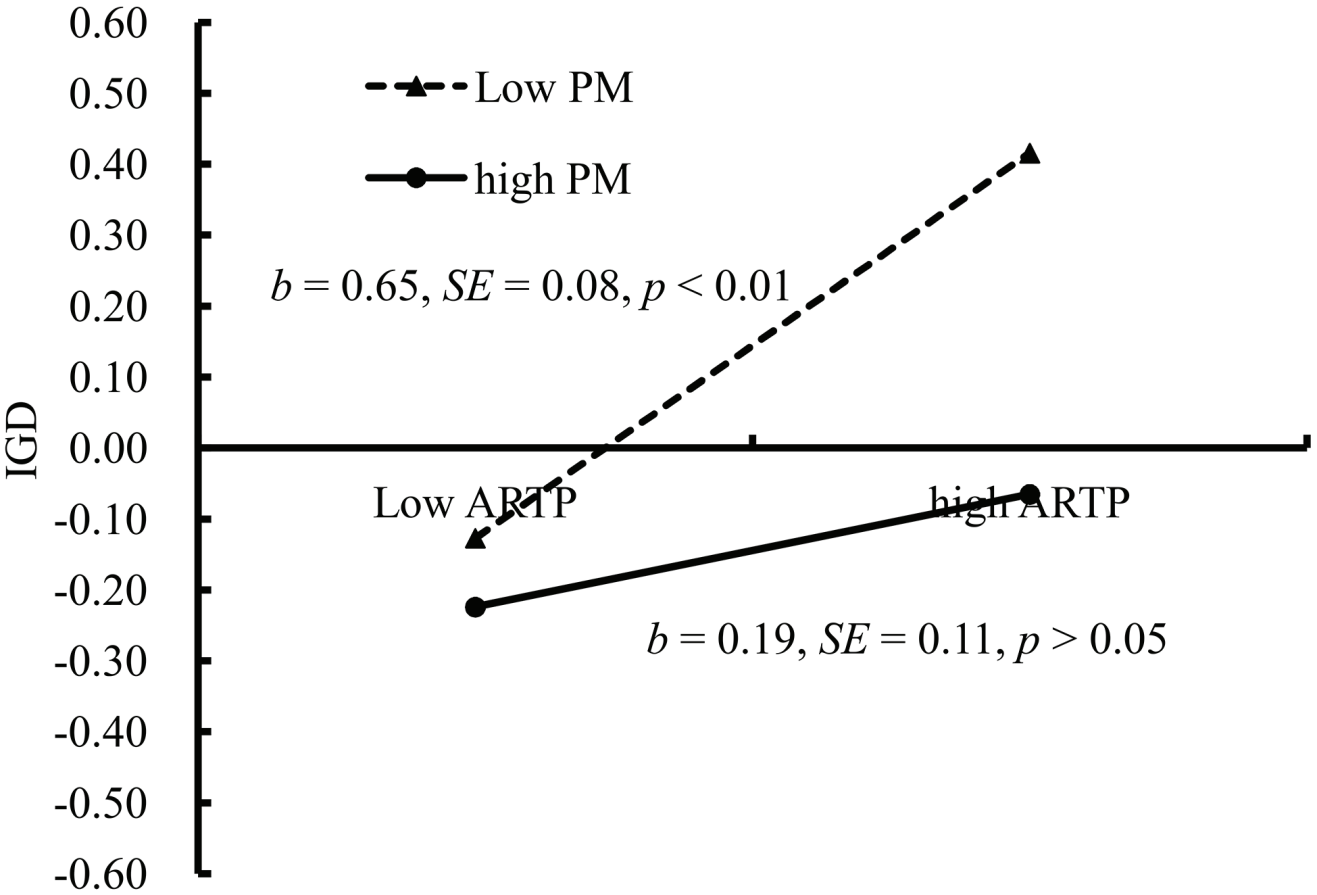
IGD among adolescents as a function of affiliations with risk-taking peer groups and parental monitoring. Note: ARTP, affiliations with risk-taking peer groups; IGD, Internet gaming disorder; PM, parental monitoring.

Affiliations with risk-taking peer groups was positively related to IGD (*b* = 0.42, SE = 0.07, *β* = 0.12, *t* = 6.10, *p* < 0.01), and this relationship was moderated by parental monitoring. Figure 6 presents the predicted scores on the IGD questionnaire as a function of affiliations with risk-taking peer groups and parental monitoring. The positive relationship between affiliations with risk-taking peer groups and IGD was significant among adolescents who reported lower levels of parental monitoring (1 SD below the *M*; *b* = 0.65, SE = 0.08, *t* = 10.93, *p* < 0.01, 95% CI [0.48, 0.81]) and nonsignificant among those who reported higher levels of parental monitoring (1 SD above the *M*; *b* = 0.19, SE = 0.11, *t* = 6.65, *p* > 0.05, 95% CI [−0.02, 0.40]). Thus, the relationship between affiliations with risk-taking peer groups and IGD was moderated by parental monitoring.

The bias-corrected percentile bootstrap method was used to examine the conditional indirect effects of ECV on IGD as a function of parental monitoring. Specifically, the indirect relationship between ECV and IGD *via* affiliations with risk-taking peer groups was significant for adolescents who reported lower levels of parental monitoring (indirect effect = 0.11, SE = 0.02, 95% CI [0.07, 0.15]) and nonsignificant for those who reported higher levels of parental monitoring (indirect effect = 0.02, SE = 0.01, 95% CI [−0.01, 0.05]). Therefore, the moderating effect of parental monitoring on the relationship between ECV and IGD among adolescents was mediated by affiliations with risk-taking peer groups.

## Discussion

The results of this study suggest that parental monitoring moderated the relationship between ECV and IGD and that this moderating effect was mediated by affiliations with risk-taking peer groups. These findings support our hypotheses.

First, the findings suggest that parental monitoring could significantly attenuate the adverse effects of ECV on IGD among adolescents. Specifically, the moderating effect was positive for adolescents, who reported high and low levels of parental monitoring; however, this effect was stronger for adolescents, whose parents were unaware of their children’s whereabouts than for adolescents whose parents were concerned about their children’s daily activities. Adolescents can be easily misguided in risky contexts because they might not possess an adequate ability to distinguish between what is right and wrong. Poor parental monitoring may promote adolescent rationalizations of their deviant behaviors (e.g., IGD). According to the risk-buffering model (Luthar et al., 2015), increased parental monitoring serves as a protective factor to mitigate the adverse effects of ECV (Lambert et al., 2005); as a result, it can alleviate IGD. Adolescents who report high levels of parental monitoring may receive good guidance and experience positive adjustment in violent community contexts. Furthermore, they may exhibit more normative behaviors, and consequently, be less likely to exhibit problem behaviors such as IGD (Parker and Benson, 2004; Chen et al., 2016). Indeed, past research findings have indicated that the relationship between risky environments and adverse outcomes for adolescents can be weakened by parental monitoring (Bacchini et al., 2011; Low and Espelage, 2014).

Second, the findings indicate that affiliations with risk-taking peer groups mediated the moderating effect of parental monitoring on the relationship between ECV and IGD among adolescents. Consistent with the risk-buffering model (Luthar et al., 2015) and social development model (Hawkins and Weis, 1985), the results of the present study suggest that adolescents who have been exposed to community violence and report low levels of parental monitoring are more likely to be affiliated with risk-taking peer groups, which in turn may promote IGD. However, this adverse effect was found to be substantially lower among adolescents who reported ECV but high levels of parental monitoring. More specifically, ECV may strengthen affiliations with risk-taking peer groups among adolescents who report low rather than high levels of parental monitoring; this may be the case because adolescents develop wrong cognitions when they are exposed to violent environments (Kimonis et al., 2011). If parents fail to monitor their adolescents, adolescents who live in violent community environments may gradually become less negative in their evaluations of deviant behaviors (Guerra et al., 2003); they may also choose to affiliate with risk-taking peer groups who seem to have less monitoring and more autonomy (Dishion et al., 2012). Conversely, high parental monitoring and guidance can weaken the negative impact of ECV on adolescent peer interactions (Low and Espelage, 2014). Indeed, a previous study has shown that high parental monitoring attenuates the adverse effects of ECV on victimization—an important predictor of affiliations with risk-taking peer groups—in adolescents (Low and Espelage, 2014).

In addition, the findings of this study suggest that parental monitoring moderated the relationship between affiliations with risk-taking peer groups and IGD among adolescents. Specifically, the adverse effects of affiliations with risk-taking peer groups on IGD were significant for adolescents who reported low levels of parental monitoring and nonsignificant for adolescents who reported high levels of parental monitoring. This finding also suggests that adolescents whose parents are unaware rather than aware of their children’s friendships report higher levels of IGD. This may be the case because adolescents may observe and imitate the problem behaviors of their risk-taking peers (Bandura, 1977; Ding et al., 2017), and they might have more opportunities to spend time on online games with inadequate parental monitoring, which could lead to IGD (Tian et al., 2019). Ample research suggests that affiliations with risk-taking peer groups are a risk factor for IGD among adolescents (Zhu et al., 2015; Xu et al., 2017). However, parents who engage in high levels of parental monitoring set rules about friend-making, reducing the likelihood of adolescent affiliations with risk-taking peer groups and the development of IGD (Tian et al., 2019).

The degree and content of parental monitoring may vary by race and culture (Li et al., 2003), and thus it is meaningful to discuss our findings from the Chinese cultural perspective. Indeed, parental monitoring is one of the important aspects of Chinese child-rearing behaviors, and a previous study indicated that the Chinese style of parenting generally includes more punishment and control (Liu et al., 2005). There is a Chinese proverb that states, “The children’s errors should be blamed to his parents’ failure to teach.” This indicates that parental monitoring, control, and punishment tend to be regarded as a way for parents to care, love, and take responsibility for their children, which originates from Chinese ancient times. In the Chinese context, effective parental monitoring not only improves adolescents’ behavior (Ye et al., 2018), but also promotes trust between parents and adolescents (Ying et al., 2015). Moreover, when children spend more time in school and establish friendships as they become adolescents, parental monitoring becomes less dependent upon control as parents give their children more autonomy (Ying et al., 2015). Parental monitoring that maintains the correct balance between control and autonomy can help Chinese adolescents avoid risky environments and achieve healthy development.

The findings of this study make it apparent that parental monitoring can serve as a protective factor by attenuating the relationship between ECV and affiliations with risk-taking peer groups, and between affiliations with risk-taking peer groups and IGD. In sum, the findings of this study extend prior research findings by providing a new perspective from which the effects of ECV, parental monitoring, and affiliations with risk-taking peer groups on IGD among adolescents can be understood. Thus, it enriches our understanding of the mechanisms that underlie the relationship between ECV and IGD among adolescents.

### Limitations and Suggestions for Future Research

The present study has several noteworthy limitations. First, because we used cross-sectional data to examine the relationships between the study variables, the causality of the emergent relationships cannot be delineated. Therefore, longitudinal designs must be adopted in future studies to extend the present findings. Second, the data that we collected were based on adolescent self-reports, which might have been influenced by common method bias. In order to overcome this limitation, future studies can use multiple sources of information (e.g., reports of community neighbors, parents, and peers) to collect data. Third, the participants in this study were from southern China. Thus, the validity of the results of this study must be verified using samples that have been sourced from other regions or countries.

### Practical Implications

This study has important implications for prevention and intervention strategies that are aimed at addressing IGD among adolescents. First, the findings reported that ECV was an early warning sign for identifying those adolescents at risk for developing IGD, which suggests that community managers should attempt to maintain safe communities and create healthy community environments. Additionally, parents and educators should provide preventive educational and psychological counseling for adolescents as a method of potentially decreasing the association between ECV and IGD (Yu et al., 2017). Second, our findings highlight the critical role that parental monitoring plays in mitigating the adverse effects of ECV on adolescents. Thus, enhancing the level of parental monitoring should be considered as a priority intervention strategy to reduce the risk of IGD among adolescents (Ang et al., 2012). Third, our results revealed that intervening adolescents’ friendships with deviant peers might be an effective strategy for reducing the possibility of adolescents developing IGD. Thus, both strategies could serve to reduce adolescent risk, as improving the community environment might reduce opportunities for risk-taking peer groups to gather, and parents and teachers could assist adolescents with identifying risk-taking peer groups and developing positive friendships to inhibit IGD. In general, reducing community violence, avoiding risk-taking peer groups, and strengthening parental monitor might contribute to the prevention of adolescent IGD.

## Data Availability

The datasets generated for this study are available on request to the corresponding author.

## Author Contributions

QL and CY conceived and designed the research, performed the research, and wrote the manuscript. QL, CY, and QC analyzed the data. QL, CY, QC, XX, HW, JX, SH, and KD revised the article critically for important intellectual content, commented on, and approved the final manuscript.

### Conflict of Interest Statement

The authors declare that the research was conducted in the absence of any commercial or financial relationships that could be construed as a potential conflict of interest.
